# The Advantages of Nitrate of Silver in Dental Practice

**Published:** 1893-02

**Authors:** A. M. Holmes


ARTICLE IV.

### THE ADVANTAGES OF NITRATE OF SILVER IN DENTAL PRACTICE.

BY A. M. HOLMES, D. D. S.

When I received the request from the President of the Sixth District Dental Society to read a paper at this meeting, I hesitated, and was at a loss to know what to contribute that had not been written and talked over and over in society meetings; still, duty required that I should respond, and I decided to give something of my personal experience in the use of nitrate of silver in the treatment of diseases of the teeth, with the hope that, although it had but recently been up for discussion in dental societies, by reason of the able papers of Dr. Stebbins, the subject had not become threadbare, and that you would find something of interest in its consideration. The character and scope of the discussions that I have read on the use of this remedy for the treatment of diseased teeth, have been such as to impress me with the belief that its benefits are not generally understood and appreciated.

Nitrate of silver is conceded to rank as one of the most efficient and reliable remedies in medicine and surgery, and when its merits are fully known it is believed that it will be found equally efficient in the treatment of a large class of diseases of teeth. Take for instance decay in temporary teeth; we all know from individual experience how trying it often is to fill the teeth of small children, in the ordinary way of making such operations; how they resist all efforts to excavate and fill sensitive cavities. By the use of nitrate of silver these operations are more easily made.

In approximal cavities in the posterior teeth, where the child is not too nervous and timid, cut away the walls to a V-shape, prepare a piece of gutta-percha of the proper size to fill the space, soften it by heat, and cover the parts that are to come in contact with the diseased surfaces with powdered crystals of nitrate of silver, and carry it to the place in the tooth or teeth prepared for its reception, packing it firmly, and leaving it there to be worn away by use in mastication. When that takes place, the surfaces of the teeth treated will be found black and hard, with no sensitiveness to the touch, or to change of temperature, and they will remain so indefinitely. In case the child is so timid and fearful as to prevent this course, dry the cavity, take out such softened dentine as the patient will permit,carry the crystals on softened gutta-percha into the cavity, and pack it, leaving it to the time when it is desired to replace it with a more thorough operation. On removal of this filling, the dentine will usually be found hard, without sensitiveness, and needing but little excavation for the final filling.

I have treated diseased pulps with nitrate of silver crystals very frequently, since early in my practice, especially in temporary teeth, where devitalizing pulps with arsenious acid is unsafe, applying the crystals direct to the exposed pulp, usually with relief to the patient.

Nitrate of silver is a resolute remedy, it cauterizes the surfaces of the soft tissues to which it is applied, but does not penetrate them as does carbolic acid, nor does it involve the entire pulp in an inflammatory condition, tending to destroy the whole mass, as does arsenious acid.

In cases of extreme sensitiveness about the necks of the teeth at the margins of the gums, where the tendency is to softening of the tissues of the tooth, a condition very annoying to the patient and troublesome to the dentist, nitrate of silver has proved more successful with me than any other remedy, in checking the progress of the disease and relieving the patient. The salt may be applied directly to the sensitive part without pain to the patient. A good method that I have practiced, is to cover the parts after the nitrate is applied with a phosphate filling material of a cream-like consistency. That hardens and prevents the washing away of the remedy, and the surrounding parts from coming in contact with the salt.

Erosion, or wasting of the teeth, is checked by nitrate of silver more perfectly than by any other remedy that I have ever used. The salt is applied to the affected parts, and covered with a phosphate filling to protect and retain it in place until it is firmly established in the dentine. In cases where the progess of the disease has gone so far as to require restoration by filling, this preliminary treatment is very beneficial in preventing a further waste of the tooth substance, and consequent failure of the operation.

In cases of superficial decay in soft teeth, where dark surfaces are not objectionable, nitrate of silver is very beneficial. By removing the softened portion of the tooth, polishing the surface and rubbing the salt into the dentine, using a warm burnisher, and varnishing the parts to protect them and to hold the remedy until it is taken into the organic matter of the tooth, there will succeed a dense, hard surface, free from sensitiveness in mastication or change of temperature. In filling cavities in the class of teeth having an excess of organic matter, with which there is so much trouble from chemical or electro-chemical action between the walls of the cavity and the filling, an application of nitrate of silver will effectively prevent these unfavorable results. The remedy is taken up by the dentine, penetrating the surface sufficiently to prevent any such action between filling and tooth.

This treatment will at times result in a darkish hue to the walls of the cavity about the filling. This I explain to patients, that they may know that it results from the treatment, and that it is a proper and favorable condition for permanency of the operation. In crowns and bridges, when the dentine is uncovered, it is beneficial to use this remedy on the teeth and roots used to sustain the bridge or crown, as a protection against thermal change, and decay. The use of nitrate of silver may be varied by applying the rubber dam, using a strong solution of the salt, and evaporating the moisture by use of a hot air syringe. When used in this way, a solution of soda can be applied to the parts to neutralize any acid remaining. In this class of cavities extending so far beneath the soft tissues as to render the use of the rubber dam or matrix impracticable, and a leakage from the surrounding tissues is liable to enter the cavity while introducing the filling and injure the permanency of the operation, cauterizing these tissues thoroughly with nitrate of silver will effectually prevent such a result.

After treatment of diseased pockets, and removal of the deposits from the roots of teeth, nitrate of silver has proved more successful in restoring a healthy condition of the parts than any other remedy that I have used in the treatment of pyorrhœa. The finely pulverized crystals may be applied by a small spatula of wood or platinum, slightly dampening the end of the instrument and applying it to the salt. The crystals will adhere sufficiently to be easily placed in the space between the gum and the roots of the teeth. After the remedy has been left for a few moments in contact with the parts, it may be washed away with water, by the use of a syringe.

In cases of the extirpation of pulps, where the canal is sensitive at or near the apex of the root, nitrate of silver crystals carried to the sensitive part and left there for a few hours usually relieve the trouble, and the canal can be filled without pain or danger of unfavorable results.

There are some of the many cases in which nitrate of silver crystals are advantageous in dental practice. I will not detain you longer, for it was not the purpose of this paper to cover the entire field of this remedy It is a powerful agent. It acts promptly, with great uniformity, and leaves its track in darkened surfaces when applied to the teeth. This should be considered, and its employment governed accordingly.—*Dental Pract. and Advertiser.*

---

ARTICLE V.

